# The SAMBA tool uses long reads to improve the contiguity of genome assemblies

**DOI:** 10.1371/journal.pcbi.1009860

**Published:** 2022-02-04

**Authors:** Aleksey V. Zimin, Steven L. Salzberg

**Affiliations:** 1 Department of Biomedical Engineering, Johns Hopkins University, Baltimore, Maryland, United States of America; 2 Center for Computational Biology, Johns Hopkins University, Baltimore, Maryland, United States of America; 3 Department of Computer Science, Johns Hopkins University, Baltimore, Maryland, United States of America; 4 Department of Biostatistics, Johns Hopkins University, Baltimore, Maryland, United States of America; Carnegie Mellon University, UNITED STATES

## Abstract

Third-generation sequencing technologies can generate very long reads with relatively high error rates. The lengths of the reads, which sometimes exceed one million bases, make them invaluable for resolving complex repeats that cannot be assembled using shorter reads. Many high-quality genome assemblies have already been produced, curated, and annotated using the previous generation of sequencing data, and full re-assembly of these genomes with long reads is not always practical or cost-effective. One strategy to upgrade existing assemblies is to generate additional coverage using long-read data, and add that to the previously assembled contigs. SAMBA is a tool that is designed to scaffold and gap-fill existing genome assemblies with additional long-read data, resulting in substantially greater contiguity. SAMBA is the only tool of its kind that also computes and fills in the sequence for all spanned gaps in the scaffolds, yielding much longer contigs. Here we compare SAMBA to several similar tools capable of re-scaffolding assemblies using long-read data, and we show that SAMBA yields better contiguity and introduces fewer errors than competing methods. SAMBA is open-source software that is distributed at https://github.com/alekseyzimin/masurca.

## Author summary

The DNA molecule that is in almost every cell in a living organism can be represented as sequence of four different nucleotides, or bases denoted by letters A,C,G, and T. The current sequencing technologies require breaking the DNA molecule into short fragments, sequencing them to find the corresponding sequence of letters, producing “reads”, and assembly, which recovered the DNA sequence from the reads. Repeats in the genome sequences typically prevented one from recovering full contiguous genome sequence because any repeat that is longer than the size of the read cannot be reliably resolved. Third-generation sequencing technologies can generate very long reads albeit with relatively high error rates. The lengths of the reads, which sometimes exceed one million bases, make them invaluable for resolving complex repeats that cannot be assembled using previous-generation reads. Many high-quality genome assemblies have already been produced, curated, and annotated using the previous generation of sequencing data, and full re-assembly of these genomes with long reads is not always practical or cost-effective. Here we introduce a tool called SAMBA that is designed to upgrade existing assemblies using additional coverage with long-read data, resulting in substantially greater contiguity. Here we compare SAMBA to several similar tools capable of re-scaffolding assemblies using long-read data, and we show that SAMBA yields better contiguity and introduces fewer errors than competing methods. SAMBA is open-source software that is distributed at https://github.com/alekseyzimin/masurca.

## Introduction

The development of long-read sequencing technologies has revolutionized the genome assembly landscape. It is possible now, for example, to get reads that are up to a million bases long from Oxford Nanopore’s MinION and PromethION instruments [1]. These "ultralong" reads are extremely helpful for assembling genomic regions filled with long, complex repeats. However, the high error rates are still an obstacle to obtaining high-quality genome sequences from long reads alone. One effective strategy for creating assemblies with long reads is to generate both short and long reads, and then to assemble the “easy” parts of the genome with short reads and use the long reads to resolve the hard-to-assemble regions. However, for many genomes that have already been sequenced, existing assemblies already capture most of the non-repetitive sequence, obviating the need to generate additional short-read data. For these genome assemblies, adding long read data might provide a fast, cost-effective way to improve their contiguity.

A hybrid approach for scaffolding genomes using long-read data was first proposed in [2]. The underlying algorithm is straightforward: first it maps long reads to contigs, then it uses the alignments to link the contigs into a graph, and finally it traverses the resulting graph to produce scaffolds, in which gap sizes are estimated from the linking information. In practice, implementing this approach may be quite complex, due to repetitive alignments that introduce loops and nested loops in the graph, and to erroneous links that result from spurious alignments of reads.

In this paper we describe a novel tool called SAMBA (Scaffolding Assemblies with Multiple Big Alignments) that uses long reads to re-scaffold contigs from an existing genome assembly and to fill gaps in those scaffolds. The tool is designed to use 10-30x coverage in long read data with a set of existing contigs, although it can function with lower or higher coverage as well. Several previously developed tools also allow for scaffolding with long reads. These include AHA, which is part of the SMRT software analysis suite [2], SSPACE-LongRead [3], and LINKS [4], all of which were designed for bacteria and other small genomes. The SMIS scaffolder (https://www.sanger.ac.uk/tool/smis/) utilizes an approach of creating artificial “mate-pairs” from the long reads and using that mate-pair information to scaffold the contigs. The recently published LRScaf tool [5] is one of the very few tools besides SAMBA that can handle mammalian-sized genome data, but its algorithm is prone to creating artificial duplications of contigs. All tools except for SMIS attempt to merge contigs when negative gaps (overlaps between contigs within a scaffold) are detected, however none of these tools utilize the consensus of the long reads to fill gaps in the scaffolds as SAMBA does.

The SAMBA tool described here is also a general-purpose assembly improvement tool. It has two modes or operation that are set by the “-d” parameter. It takes as input an assembly, which can be a set of contigs or scaffolds, plus either a set of long reads (“-d ont” for ONT reads, or “-d pbclr” for PacBio CLR or legacy reads) or another set contigs from the same or a closely related genome, or PacBio HiFi reads (“-d asm”). It then uses the reads or the other contigs to link and merge contigs of the input assembly. SAMBA is fast and memory efficient: it can scaffold and gap-fill a mammalian genome in only a few hours on a modern 24–32 core server, utilizing no more than 50Gb of RAM for a human-size genome. In this paper we present the results of running SAMBA on data sets from *Arabidopsis thaliana* and human, and we compare its accuracy and run times to other comparable recently published and currently maintained software.

We note that in cases where the original assembly was built from only short-read data, full re-assembly of the genome using both long and short reads, with a hybrid assembler such as MaSuRCA [6,7] or Wengan [8], will likely offer better results than re-scaffolding with SAMBA. However, in many cases full re-assembly is not practical due to resource constraints or to the unavailability of the original data. Also for existing assemblies that already used some long read data, post-processing with even longer reads with SAMBA will yield improvements at a low computational cost.

SAMBA is open source software, distributed with the MaSURCA assembler, included in version 4.0.5 and above.

## Results

We compared the performance of SAMBA with three other long-read scaffolding algorithms: LRScaf version 1.1.11 [5], LINKS 1.8.7 [4], and SMIS v0.1-alpha (https://www.sanger.ac.uk/tool/smis/). Because AHA and SSPACE were designed for bacterial genomes, their memory (RAM) requirements make them impractical to use for large (mammalian-sized) genomes, so we did not include them in these experiments.

For the experimental comparisons, we used data sets from two organisms, *Arabidopsis thaliana* Ler-0 and human, see Table 1. The Illumina and PacBio data for Arabidopsis was described in [9], and the high-quality assembly of these data is available from [10]. See Data Availability section for more details, including the URLs and accession numbers.

**Table 1:** Data used for the scaffolding experiments, including short and long reads from the model plant *Arabidopsis thaliana*, and reads from two different individual humans. The Illumina reads were paired-end reads of a fixed length. N50 read length is computed by summing all read lengths, and determining the value N50 such that 50% of that total length is covered by reads of N50 or longer.

	Number of reads	Genome coverage	N50 read length
***A*. *thaliana***			
**Illumina**	46,129,480	115x	300
**PacBio P6-C4**	3,448,228	118x	7,205
***H*. *sapiens* CHM13**			
**Oxford Nanopore**	29,468,868	118x	56,695
***H*. *sapiens* HG01243**			
**Illumina paired-end**	1,307,137,030	59x	150
**PacBio CLR**	23,672,554	113x	28,837
**Oxford Nanopore**	16,239,849	58x	44,052

### *Arabidopsis thaliana* evaluation

We first assembled the genome of *Arabidopsis thaliana* from Illumina paired-end reads, without using PacBio data, using MaSuRCA 4.0.3 [6,7]. For clarity we will refer to this assembly as Athal-Illumina. The Athal-Illumina assembly contained a total of 118,048,454 bp of sequence in 4663 contigs, with an NG50 contig size of 172,147 bp; i.e., the size at which >50% of the assembly is contained in contigs of this length or longer. The NG50 size was computed by Quast [11] using as reference the previously-published assembly of the same plant produced with the PacBio data [10], which we refer to as Athal-Berlin. We did not use the Arabidopsis reference genome for this comparison to avoid introducing noise due to possible structural differences between the reference plant and the plant used for sequencing and assembly of Athal-Berlin. NG50 differs from the standard N50 computation in that the genome size used for the computation is that of the reference (Athal-Berlin) rather than that of Athal-Illumina itself. Further evaluation of the Athal-Illumina assembly with Quast reported an NGA50 of 150,736 bp with 139 mis-assemblies. Quast computes NGA50 by splitting the query (Athal-Illumina) genome at mis-assemblies detected by aligning it to the reference (Athal-Berlin), and then re-running the NG50 computation.

Note that in general, SAMBA is indifferent to the choice of the assembler used to produce the input contigs, as long as the contigs satisfy our size guidelines, including an N90 value larger than the minimum alignment length parameter.

We then scaffolded Athal-Illumina using (1) additional PacBio reads and (2) the Athal-Berlin assembly. For the first re-scaffolding, we randomly chose multiple subsets of long PacBio reads, each representing 10-50x coverage, from the total of 118X coverage available (Table 1). We then used these to re-scaffold the Athal-Illumina assembly, and then to evaluate the scaffolder’s performance as function of long-read coverage. We evaluated assembly quality with Quast before and after scaffolding, using the Athal-Berlin assembly as the reference. For both SAMBA and LRScaf we used similar scaffolding parameters, setting the minimum long-read alignment length to 2500 bp and the maximum overhang length to 1000 bp. We experimented with different values of minimum alignment length, and 2500 seemed to provide the best balance between the number of mis-assemblies and the size of output scaffolds for this data set. Decreasing that number led to more aggressive scaffolding (i.e., larger scaffolds) at the expense of a higher mis-assembly rate for both programs. LINKS and SMIS use different approaches to scaffolding and do not offer the user the ability to adjust these parameters, and thus we used the defaults for these programs.

**Fig 1:**
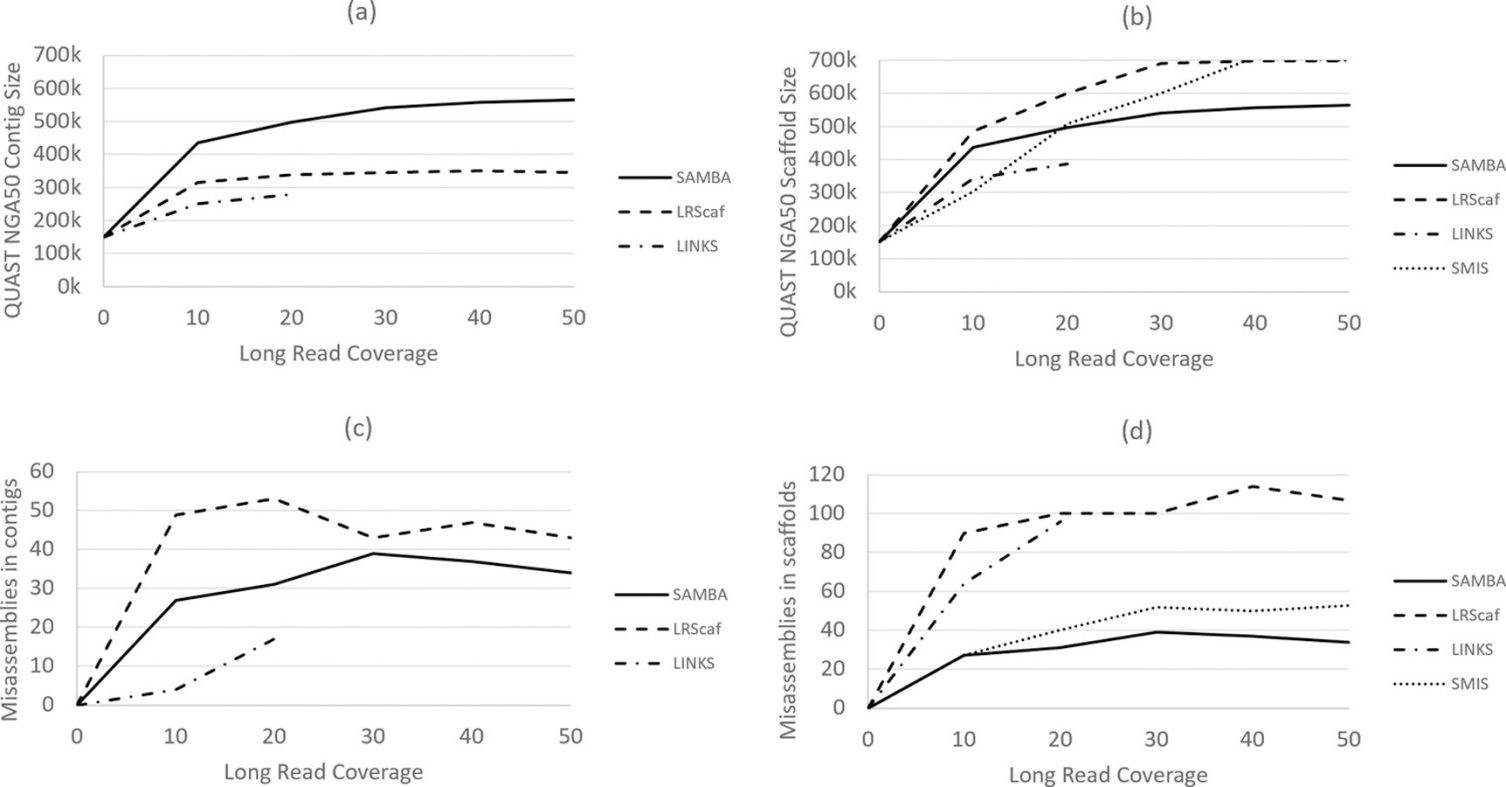
Scaffolding the Athal-Illumina assembly with LINKS, SMIS, LRScaf, and SAMBA. We compared performance as a function of long-read (PacBio) coverage, shown on the x-axis. Panels (a) and (b) show the NGA50 size for contigs and scaffolds after scaffolding with each tool. SAMBA outputs contigs, and thus its scaffold and contig results are the same. Panels (c) and (d) show the number of mis-assemblies introduced by the scaffolding process. The LINKS scaffolder required more than 512GB of RAM for PacBio coverage >20x, and thus we did not run it with higher coverage. SMIS does not change contigs, so we did not include its statistics on panels (a) and (c).

The scaffolding results for Arabidopsis as a function of long-read coverage depth are shown in Fig 1. SAMBA had the smallest number of scaffolding errors (Fig 1D), and it yielded the longest contigs (Fig 1A). LRScaf and SMIS created the longest scaffolds (Fig 1B). LRScaf had the highest number of errors in both contigs and scaffolds (Fig 1C and 1D). Improvements in contiguity obtained by all scaffolders saturated at about 30x coverage by long reads. We also noticed that the total amount of sequence in LRScaf’s output varied substantially for different inputs, ranging from 118–127 Mbp for this 120 Mbp genome. We traced that to a tendency for LRScaf to create erroneous duplicate copies of contigs on the ends of some scaffolds. (Note that we reported this as an issue on the LRScaf github site.)

When we used the Athal-Berlin assembly [10] to scaffold the Athal-Illumina assembly, we were able to achieve an NGA50 of 1,173 Kbp, more than twice as large as the 567 Kbp that was the best NGA50 length when we re-scaffolded with long reads alone. Not surprisingly, assembly using Illumina and PacBio data was far superior to using Illumina data alone, so it would make more sense to simply use both data sets in the first place. However, if one is assembling a genome that is very closely related to a previously-assembled species, one can use this approach to improve an Illumina-only assembly. SAMBA introduced only one contig mis-assembly, compared to 30 or more mis-assemblies introduced when using the PacBio data with any of the scaffolding programs (Fig 1D). This shows the effectiveness of SAMBA in reconciling different assemblies of the same genome. Ideally, we would like to achieve the same contiguity as the reference when scaffolding with a better assembly of the same species, but contig mis-assemblies in the Athal-Illumina assembly prevented that here. Below we discuss further the issue of detecting contig mis-assemblies.

We evaluated how the quality of consensus of the scaffolded assembly varied with the coverage by the additional long read data. For this evaluation we used the mismatches and insertion/deletion statistics computed by Quast before and after running SAMBA. We then computed the number of total bases added to the assembly in “patches” (gap-filling sequence) and computed the average error rate of the patches. The overall error rate for the Arabidopsis patches was ~5%, as shown in Table 2. The PacBio data used for this experiment had error rate of about 17%, which we estimated by mapping the reads to the Athal-Berlin assembly with minimap2. The error rate in the patches only decreased very slightly as the coverage increased, primarily because higher coverage allowed SAMBA to span a larger number of gaps, but not necessarily with higher coverage of the patches.

**Table 2:** Evaluation of the completeness and the consensus quality of the Arabidopsis assemblies after scaffolding with PacBio data with SAMBA. We computed the average consensus quality of the gap-filling sequences for different levels of long-read coverage based on the number of mismatches, insertions and deletions reported by QUAST before and after scaffolding. Because more gaps were closed at higher coverage, the average error rate of the patches decreased only marginally.

Additional long read coverage	10x	20x	30x	40x	50x
Gaps filled	741	831	874	907	910
Bases in patches that filled gaps	312,587	378,735	425,150	516,455	548,622
Average error rate for the patches	6%	5%	5%	4.8%	4.5%

Evaluation of the Athal-Illumina assembly with Merqury [12] yielded a quality value (QV) of 51, and the re-scaffolded assemblies output by SAMBA had QVs ranging from 42 to 43 for 10x-50x coverage of PacBio reads. SAMBA-scaffolded assemblies can be further polished, increasing the overall consensus quality substantially; for example, we polished the 30x re-assembly with POLCA [13] using the Illumina reads, and improved the QV to 57, corresponding to fewer than 5 errors per megabase.

We observed that, due to the sequence added in the gaps, the completeness of the assemblies after scaffolding with SAMBA increased slightly, from 93.75% in the original Athal-Illumina assembly to 94% in the assembly scaffolded with SAMBA using 50x coverage in PacBio data.

### *Homo sapiens* evaluation

Next, we examined the scaffolders’ performance on human data, which we evaluated on two different human data sets. The first data set is from CHM13, a haploid human cell line that was used to create the first-ever complete human genome assembly [1,14], and the second data set is diploid data from a Puerto Rican individual, HG01243, sequenced as part of the human pangenome project (see Data Availability section). Details of these datasets are listed in Table 1. The Telomere-to-Telomere (T2T) consortium has produced a very high-quality, gap-free assembly of CHM13 called CHM13 v1.1 [1] which makes evaluations of assembly correctness for the CHM13 data straightforward. However, due to its pseudo-haploid nature, the CHM13 genome is considerably easier to assemble than a typical diploid human. Thus we also include in our comparison assemblies of the diploid human sample HG01243.

For the first human comparison, we used a draft assembly of CHM13 produced by the Genome Institute at Washington University School of Medicine in 2015 (NCBI accession GCA_000983455.2). This assembly, which we refer to as CHM13-WashU, was produced with the Falcon assembler using 52x coverage in PacBio reads. Evaluation of this assembly with Quast yielded an NGA50 contig size of 9,287,872 bp, with 2,346 apparent mis-assemblies when compared to the CHM13 v1.1 assembly. To re-scaffold the CHM13-Washu assembly, we used the ONT reads from the CHM13hTERT cell line described in Table 1.

For the comparison on diploid human data, we produced an assembly of HG01243 from Illumina and PacBio data (Table 1) using MaSuRCA, which we refer to as HG01243-IP. For comparisons, we used the recently-published complete genome of HG01243, called PR1 [15] as the reference in QUAST. Evaluation of the HG01243-IP assembly with yielded an NGA50 contig size of 5,839,185 bp with 1,183 apparent mis-assemblies when compared to PR1.

We then used SAMBA, LINKS, SMIS and LRScaf to scaffold the CHM13-WashU and HG10243-IP assemblies using randomly chosen subsets of ultralong ONT reads representing 10x, 20x and 30x coverage. We set the minimum long read alignment to 5000 bp for both SAMBA and LRScaf. LINKS required more than 512 GB of computer memory even at the lowest level of ONT read coverage, and SMIS was unable to complete the scaffolding in 2 weeks on a 24-core server; therefore, we did not include the results from either of these tools in these comparisons. We evaluated contiguity and correctness with Quast using CHM13 v1.1 as the reference.

**Fig 2:**
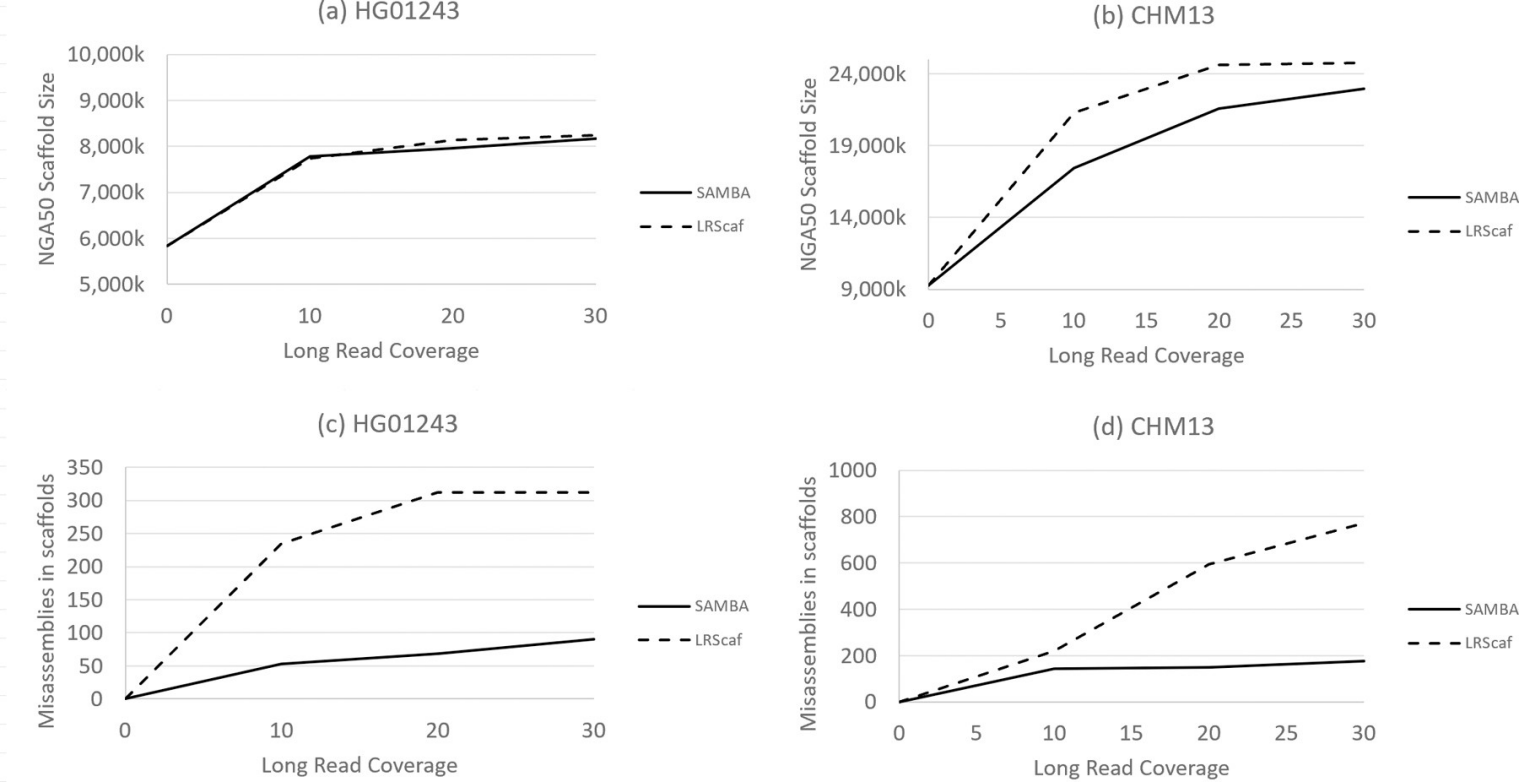
Contiguity and accuracy results for SAMBA and LRScaf on scaffolding of human assemblies CHM13 (CHM13-WashU) and HG01243 (HG01243-IP) using varied coverage of Oxford Nanopore Technologies (ONT) ultralong reads. The x-axis shows coverage in the ONT reads. All assemblies were evaluated by Quast using the CHM13 v1.1 assembly as the reference for CHM13, and the PR1 assembly (Zimin et al., 2021) for HG01243. Panels (a) and (b) compare NG50 sizes for the two different human assemblies, while (c) and (d) compare the number of mis-assemblies. Both scaffolders produced similar NG50 sizes for HG01243, while LRScaf produced bigger scaffolds for CHM13 data. SAMBA had fewer mis-assemblies in both genomes at all depths of coverage.

Fig 2 compares the resulting scaffold sizes as a function of read coverage for both assemblies, using NGA50 size for CHM13 and HG01243. Panels (a) and (c) refer to HG01243 evaluations and panels (b) and (d) refer to CHM13 evaluations. For the CHM13 genome, LRScaf produced bigger scaffolds with more errors than SAMBA, while for the HG01243 assembly SAMBA contigs were as big as scaffolds produced by LRScaf, while having much fewer mis-assemblies.

In terms of cost-benefit analysis, we observe that scaffold sizes continued to increase as coverage in ONT reads rose from 20x to 30x, suggesting that even deeper coverage would yield some additional improvements.

For the human experiments we used the ONT reads to create the final consensus of the gap-closing sequences, or patches. As mentioned above, the ONT reads in our experiments had a lower error rate than the PacBio reads. This difference resulted in a higher consensus quality for the patches, ranging from an average of 3.5% error for the CHM13 assembly with 10x in additional ONT coverage down to 2.6% with 30x ONT coverage.

In all our experiments, SAMBA ran much faster than SMIS and LINKS, but somewhat slower than LRScaf. For example, at 30x coverage in the Arabidopsis experiment (Fig 1), SAMBA scaffolded the Athal-Illumina assembly in about 36 minutes, with 30 minutes spent on computing consensus on the sequence “patches” used for merging contigs. On the same data set, LINKS took nearly 10 hours and SMIS took about 28 hours (Table 3). LRScaf was the fastest, completing the scaffolding in under 5 minutes, with most of the time (4 minutes) spent in alignment of the long reads with minimap2. SAMBA does extra work, as compared to LRScaf, in computing the consensus to fill the gaps. When the time spend computing the gap sequences is subtracted, LRScaf and SAMBA have very similar run times. On the human data (Fig 2), SAMBA completed scaffolding and gap filling with 30x Nanopore long reads in 12 hours, with 9 hours spent on initial read alignment by minimap2 and 3 hours spent on computing the consensus sequences to fill gaps. LRScaf finished the scaffolding in about 9 hours. The time spent on scaffolding (resolving the contig graph) was only a few minutes for both LRScaf and SAMBA. For all the timings reported here, we used a 24-core Intel(R) Xeon(R) 6248R server with 512 GB of RAM. SAMBA was quite memory -efficient, using at most 50GB of RAM for the highest long-read coverage human experiment, and less than 2GB or RAM for the Arabidopsis experiment with 50x long-read coverage.

**Table 3:** Total wall clock time (in hours) that each tool required for scaffolding the *Arabidopsis* and human assemblies with 30x coverage by long reads on a 24-core Intel(R) Xeon(R) 6248R server with 512 GB of RAM. In parentheses we list the time required for the alignment of the long reads for SAMBA and LRScaf.

	SAMBA	LRScaf	LINKS	SMIS
*A*. *thaliana*	0.6(0.07)	0.1(0.07)	10	28
Human (CHM13)	12(9)	9(8.9)	--	--

## Design and implementation

The input to SAMBA comprises a set of input contigs C and a set of sequences used to join the contigs together, which we will designate as J. J can contain either long reads or contigs from another assembly of the same organism made, for example, using a different assembler. SAMBA starts off by aligning all sequences J_i_ in J to the contigs C_k_ in C using minimap2 [16]. We only use the best alignments that are longer than 5Kb (a parameter) that may overhang the end of the contig by no more than 1Kb (also a parameter), and we call these the "proper" alignments. An overhang occurs when an alignment of sequence C_k_ to a sequence J_i_ is such that the alignment stops before an end of C_k_, but J_i_ continues beyond the end of C_k_. The reason why we allow for overhangs is that contigs may have local mis-assemblies on the ends, which frequently are the reason why an assembler could not extend the contig. SAMBA can sometimes fix these mis-assemblies while merging contigs with long reads. The requirement of a minimum alignment length ensures that short repeat-induced overlaps are filtered out. We only keep the proper alignments of sequences in J that align to two or more contigs in C, and discard all alignments where J_i_ only aligns properly to a single contig. We note here that in practice SAMBA performs better on relatively contiguous input assemblies, with an N90 >10 Kb; i.e., 90% of the assembled sequence is found in contigs of 10 Kb or longer.

A proper alignment of a sequence J_i_ to two contigs C_i_ and C_j_ in C creates a proper edge between C_i_ and C_j_. If the set J consists of reads that represent relatively deep coverage, we require more than one proper edge between each pair of connected contigs. We then bundle the proper edges and produce consensus sequences for them using the Flye assembler’s consensus module [17]. This results in a set of linking sequences. If J represents another assembly of the same or a similar genome (as opposed to a set of long reads), then we skip the consensus step. We then re-align the linking sequences to the contigs in C with minimap2 and build a graph of contigs in C connected by the proper edges, where the edges are the proper alignments of the linking sequences. Each node (each contig) naturally has a beginning and an end with an arbitrary (randomly chosen) direction, and we designate all adjacent edges as either incoming or outgoing.

**Fig 3:**
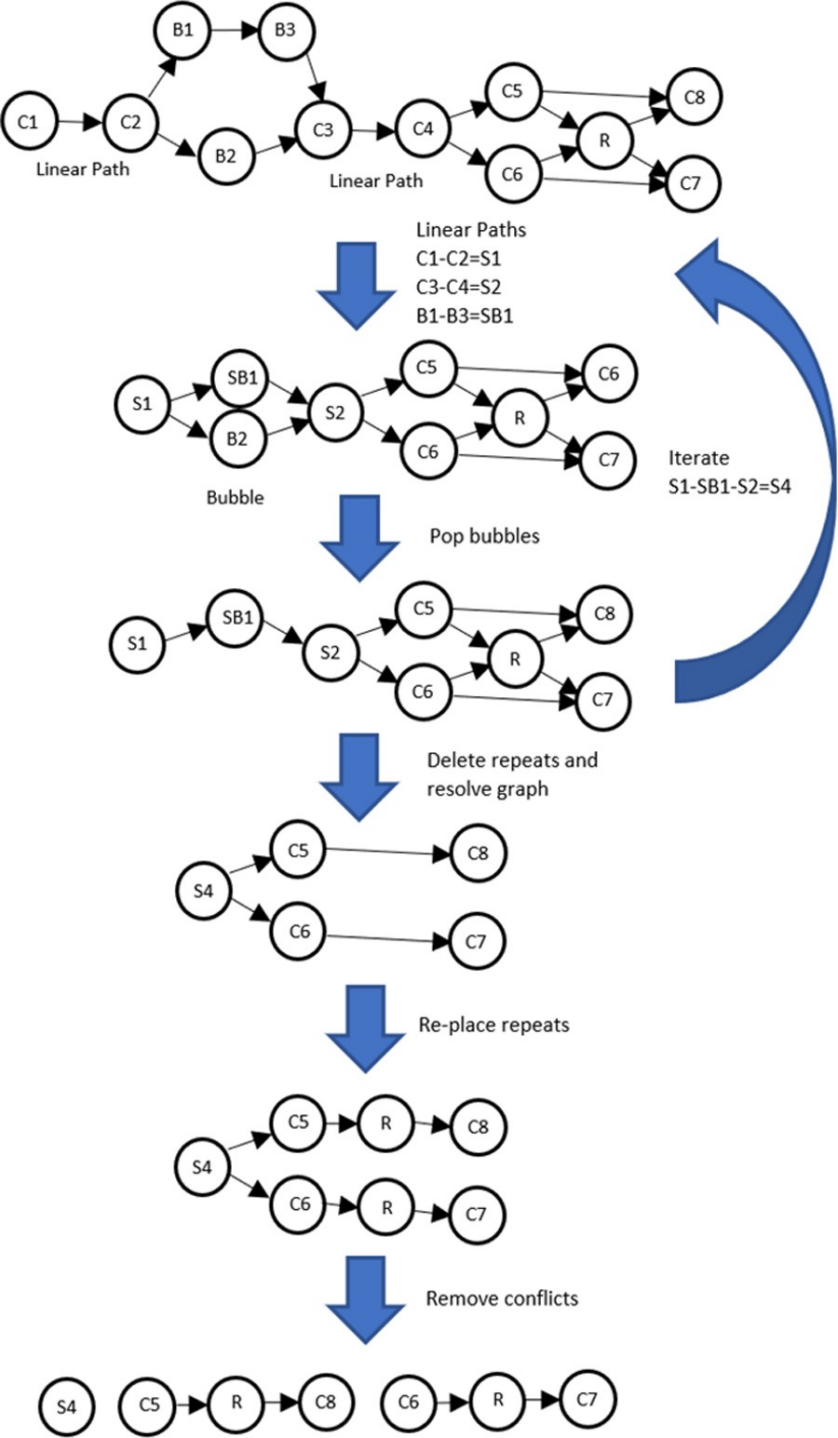
Overview of the SAMBA scaffolding algorithm. The method resolves the initial graph into three final contigs, C1-C2-B1-B3-C3-C4 (S4), C5-R-C8 and C6-R-C7. The repeat contig R is duplicated in the final output. Note that if there were no proper edge between contigs C5 and C8, then the final output would contain C5 and C8 standalone instead of C5-R-C8.

The next step is to search for linear paths in the graph, defined as sequences of nodes that have exactly one incoming and one outgoing edge. We collapse such paths into super-nodes, as shown in Fig 3. Following this step, we search for "bubbles," which are paths that branch into two alternative paths and then re-merge. We call the nodes in the bubbles B_1_…B_n_, where n> = 2, and all incoming edges to B_1_…B_n_ are outgoing from the same node C_i_ and all outgoing edges are incoming to the same node C_j_. For example, Fig 3 shows a bubble beginning at node C_2_, which has two outgoing edges, and ending in node C_3_, with two incoming edges. A common source of these bubbles is a haplotype variant in the original diploid genome, which may lead to a contig graph where two haplotypes representing the same location on the genome were assembled into two different contigs. We “pop” the bubbles by choosing the path through the bubble that is the longest of all B_n_ nodes. To resolve “bubbles within bubbles” we perform bubble popping iteratively until no bubbles are left to pop in the graph.

Following bubble popping, we look for repetitive contigs, indicated by nodes or super-nodes that have two or more incoming and two or more outgoing edges. In SAMBA we only aim to resolve repeats that are completely spanned by long reads in J that in turn have long overlaps with unique sequence surrounding the repeat. Therefore, we temporarily remove all repeat nodes from the graph, noting their edges, and re-compute the graph without them. This usually simplifies the graph, introducing breaks in places where there are not enough data to resolve repeats. We then examine all nodes in the resulting graph, and if a node has two or more incoming edges, we delete them both. We do the same if a node has two or more outgoing edges. Thus, for example, if a node has two outgoing edges and a single incoming edge, we keep the single incoming edge and delete the two outgoing edges. This breaks the graph into linear paths. Finally, we re-insert all repeat nodes (contigs) that we temporarily removed into the resulting linear paths according to their edges, duplicating the sequence if necessary. The final linear paths represent the scaffolded contigs. An example of the scaffolding process is depicted in Fig 3. Finally, we collapse the linear paths, and fill positive-sized gaps with the linking sequence obtained from the consensus of linking sequences in J.

Following the scaffolding and gap filling steps, we extract all patch sequences that SAMBA used to fill gaps in the final scaffolds. We use minimap2 to align all contigs in C that are shorter than the maximum patch size to the patches. We then look for contigs that are uniquely contained in a patch, aligning with more than 95% identity over more than 95% of their length. We then replace the consensus of the patches by the consensus of the contigs based on the alignment coordinates, and remove the contigs that were merged from the final output. This improves the accuracy of the patch sequences and ensures that we do not spuriously retain short contigs that are duplicates of sequences that are now present in the patches.

## Availability

SAMBA is open-source software that is distributed with free open source MaSuRCA assembly and analysis package at https://github.com/alekseyzimin/masurca.

## Future directions

We have shown here that SAMBA is a highly effective tool for improving the contiguity of genome assemblies with additional long read data. The primary limitation of SAMBA is determined by the contig lengths in the assembly being scaffolded. Since the default minimum overlap for long reads is 5000 bp, any contigs that are shorter than this minimum will not be scaffolded by SAMBA. We recommend applying SAMBA to assemblies where most of the sequence is in contigs that are longer than 5000 bp, which is not difficult to achieve with modern sequencing technologies and assembly software.

Our future plans for SAMBA development include detection and correction of contig mis-assemblies in the input assembly. We may be able to use discordant alignments, such as cases where long reads map to the interiors of two different contigs, to detect and break mis-assembled contigs. This may allow SAMBA to produce a re-scaffolded genome that is not only more contiguous, but also more accurate than the original assembly.
